# Plasma chemokines indicate enhanced bleeding in patients with chronic coronary syndrome undergoing percutaneous coronary stenting

**DOI:** 10.1007/s00392-025-02675-8

**Published:** 2025-05-21

**Authors:** Tobias Harm, Shqipdona Lahu, Katharina Mayer, Dominik Rath, Tobias Geisler, Karin Anne Lydia Müller, Marion Janisch, Kristin Adler, Götz Münch, Steffen Massberg, Adnan Kastrati, Meinrad Paul Gawaz

**Affiliations:** 1 Department of Cardiology and Angiology, University Hospital Tübingen, Eberhard Karls University Tübingen, Otfried-Müller-Str. 10, 72076 Tübingen, Germany; 2 Deutsches Herzzentrum München, Klinik für Herz- und Kreislauferkrankungen, Technische Universität München, Munich, Germany; 3 German Center for Cardiovascular Research (DZHK), Partner Site Munich Heart Alliance, Munich, Germany; 4 Department of Cardiology, Munich University Clinic, Ludwig-Maximilian University of Munich, Munich, Germany; 5 AdvanceCOR GmbH, Martinsried, Germany

**Keywords:** Antiplatelet treatment, Bleeding, Chemokine signalling, Coronary artery disease, Soluble glycoprotein VI

## Abstract

**Background:**

Patients with coronary artery disease (CAD) are at increased risk of developing ischemic events and contemporary antiplatelet therapy often leads to bleeding events following percutaneous coronary intervention (PCI). Glycoprotein VI (GPVI) is the key receptor of collagen-dependent thrombus formation and crucial for platelet homeostasis.

**Methods:**

We analysed the influence of GPVI inhibition with revacept in a randomized double-blinded trial enrolling 334 patients with CAD undergoing elective PCI. Ex vivo platelet function analyses were assessed alongside plasma chemokine concentrations. We then elucidate changes of GPVI-dependent chemokine concentrations in patients with bleeding events during the 30-day clinical follow-up.

**Results:**

Changes in platelet function occur in patients with revacept treatment and are associated with a characteristic alteration of circulating chemokine concentrations. Further, patients with adverse bleeding events share a distinct fingerprint of chemokines that is associated with modulation of in vitro platelet functions. In addition, assessment of GPVI-associated changes in chemokine signalling and platelet functions demonstrated an increased diagnostic value in patients with CAD and might improve early risk discrimination for bleeding events.

**Conclusion:**

The composition of platelet-derived chemokines correlated with platelet functions following antiplatelet treatment. Thus, assessment of chemokines may offer the perspective to identify patients at increased risk for bleeding events. Likewise, modulation of platelet chemokines in patients with revacept treatment contributes to the efficacy of antiplatelet treatment and might attenuate pathophysiological cascades leading to haemorrhagic diathesis in patients with CAD.

**Graphical abstract:**

Study design and rationale: patients with coronary artery disease (CAD) undergoing elective percutaneous coronary intervention (PCI) and the impact of treatment with soluble GPVI inhibitor revacept. Changes in ex vivo platelet function and plasma chemokine concentrations were associated with an increased bleeding risk during the clinical follow-up. Further, GPVI-associated changes in chemokine signalling might attenuate pathophysiological cascades leading to haemorrhagic diathesis and improve early risk discrimination for bleeding events

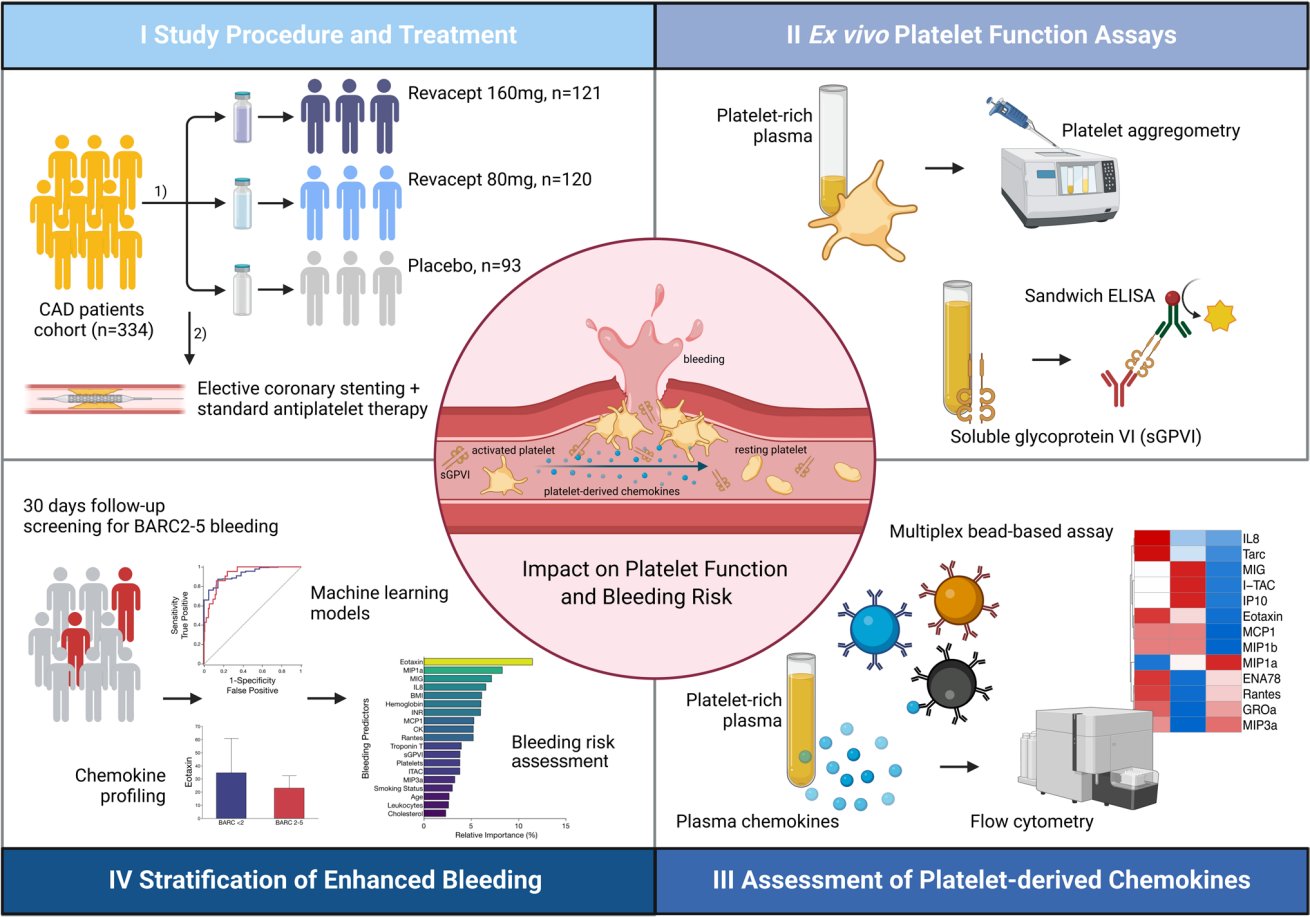

**Supplementary Information:**

The online version contains supplementary material available at 10.1007/s00392-025-02675-8.

## Introduction

Platelets play a major role in haemostasis and thrombosis [1]. Platelet hyperreactivity drives atheroprogression leading to adverse cardiovascular events [2]. Upon plaque rupture and exposure of collagen at a vascular damage site, platelet activation initiates thrombus formation [3]. Platelet glycoprotein GPVI (GPVI) binds to subendothelial collagen and promotes platelet-dependent intravascular thrombus formation leading to acute vessel occlusion [4]. Alongside promoting acute atherothrombosis, platelet adhesion and activation at the site of thrombus formation trigger chronic inflammatory cascades crucial for thrombo-inflammation and immuno-thrombosis [5]. In turn, an inflammatory microenvironment modulates platelet function and thrombus formation. Only recently, we and others unveiled that platelet function is highly dependent on circulating chemokines interacting with platelet surface receptors (thrombo-inflammation) [6, 7]. The interaction of platelets with chemokines promotes and attenuates platelet activation, depending on the chemokines present in the microenvironment of platelet-dependent thrombus formation [8].

Previously, we and others have shown that expression of GPVI on the surface of circulating platelets (pGPVI) and plasma levels of soluble GPVI (sGPVI) are able to discriminate between chronic (CCS) and acute coronary syndrome (ACS) [9, 10]. GPVI levels of circulating platelets (pGPVI) are associated with poor prognosis in patients with CAD [11]. Plasma levels of sGPVI reflect enhanced platelet activation and hyporeactivity of residual circulating platelets [12]. Recently, we found that enhanced levels of sGPVI correspond with an increased bleeding risk after PCI [13].

Limited data are available regarding plasma chemokines, sGPVI, ex vivo platelet aggregation, and the risk of bleeding following PCI in patients with CCS and treatment with dual antiplatelet therapy (DAPT). Therefore, we aimed to investigate changes in chemokine levels of patients with CCS undergoing PCI. Subsequently, we elucidate alterations in chemokine concentrations associated with antiplatelet treatment including the collagen-inhibitor revacept and investigate the potential impact on plasma chemokine signature on bleeding.

## Methods

### Study population

In this study, we included three hundred thirty-four (334) patients of the randomized multicentric research trial “Lesion Platelet Adhesion as Selective Target of Endovenous Revacept in Patients with Chronic Coronary Syndrome” (ISAR-PLASTER; Rev/CAD/02 EudraCT Number: 2015-000686-32) (Supplementary Fig. S1) [14, 15]. All patients underwent percutaneous coronary intervention for symptomatic chronic coronary syndrome (CCS) and were treated with standard DAPT with or without GPVI antagonist revacept in a double-blinded manner. According to standardized protocols, *n* = 121 patients were allocated to receive 160 mg revacept, *n* = 120 patients received 80 mg revacept, and 93 patients were allocated to receive placebo prior to PCI. Additionally, all patients were treated with antithrombotic therapy according to current international guidelines including periprocedural administration of heparin or bivalirudin, alongside antiplatelet therapy with aspirin and clopidogrel. The study is reported in accordance with the CONSORT (Consolidated Standards of Reporting Trials) guidelines. The study was approved by the local ethics committees (410/2018 AMG2) for every participating site and all patients gave written informed consent. The experiments were performed in accordance with the highest ethical standards as laid down in the Declaration of Helsinki.

### Study design

The study design has been outlined comprehensively in the initial study report and is outlined in the Supplementary methods section and Supplementary Fig. S1 [14, 15]. Inclusion and exclusion criteria of the study are summarized in Supplementary Table S1.

### Platelet function analysis

To test, whether platelet function is affected by circulating chemokines and underlies postinterventional changes in patients undergoing PCI, we performed ex vivo platelet function analysis. According to standardized protocols in vitro platelet impedance aggregometry was analysed using Multiplate Analyzer (F. Hoffmann-La Roche Ltd., Basel, Switzerland). Therefore, whole blood samples of patients enrolled into this study were collected prior to PCI and antiplatelet treatment or study drug administration (t_0 h_) as well as 48 h (t_48 h_) after PCI and allocation of study medication. Hirudinized whole blood (300 µl) of patients with CCS were stimulated with 200 µM adenosine diphosphate (ADP), as well as concentrations of 31, 93, and 253 µM/mL collagen. Platelet impedance was assessed for 6 min and results of the tests were quantified as area under the curve (AUC = AU x min) of aggregation units (AU) as previously described [14].

### Assessment of circulating chemokine and sGPVI levels

To investigate whether systemic inflammation is associated with modulation of ex vivo platelet function and the periprocedural bleeding risk in patients with CAD, we performed multilevel ex vivo function assays. Therefore, blood samples were collected at baseline (t_0 h_) before antiplatelet treatment and 48 h after PCI and administration of study medication (t_48 h_). Plasma levels of chemokines were measured using a LEGENDplex^™^ kit with specific bead-based monoclonal antibodies against individual chemokines (e.g., CXCL8 (IL8), CXCL10 (IP10), CCL11 (Eotaxin), CCL17 (Tarc), CCL2 (MCP1), CCL5 (Rantes), CCL3 (MIP1α), CXCL9 (MIG), CXCL5 (ENA78), CCL20 (MIP3 α), CXCL1 (GROα), CXCL11 (ITAC), CCL4 (MIP1ß)). Therefore, plasma of CPDA citrated whole blood samples was processed through a flow cytometry proinflammatory chemokine panel (13-plex) according to the manufacturer's instructions (BioLegend, San Diego, USA) and concentrations were reported as pg/ml. All samples were measured in duplicates and mean values were integrated into further statistical analyses. Divergence of chemokine levels before and after administration of study medication were derived from percent change of distinct chemokine concentrations (t_48 h_−t_0 h_:t_0 h_*100). At the time of baseline, sGPVI concentration was assessed as previously described [13]. Thus, a sandwich ELISA with two monoclonal rat antibodies targeting extracellular GPVI was performed to specifically measure only the soluble domain of GPVI (sGPVI). sGPVI was determined by a defined standard curve of recombinant sGPVI and concentrations are denoted by ng/ml.

### Statistical analysis

Patients baseline characteristics and platelet functional data were analysed using R (R foundation for Statistical Computing, Vienna, Austria) and JMP Pro (SAS Institute, Cary, USA). All datasets were tested for normal distribution using the Shapiro–Wilk normality test. Since all normality tests returned negative, continuous data are presented as median and interquartile range (IQR). Mann–Whitney U was performed for two group comparisons for non-normally distributed continuous variables and Kruskal–Wallis test with Dunn’s post-hoc test was applied for multiple comparisons. Categorical parameters are presented as counts and proportions and were compared using the Chi-squared test. Correlation data is based on Spearman’s rank correlation coefficient (q) and was adjusted for multiple comparisons using a false discovery rate (FDR) controlling procedure to correct significance levels (*p* < 0.05) where applicable. To identify factors associated with an increased bleeding risk after the application of revacept or placebo in patients with CAD, we utilized machine learning with extreme gradient boosting (XGBoost). Therefore, the data of the patient cohort was randomly split into a training dataset (75%, *n* = 250) and a test dataset (25%, *n* = 84) for the final model. Included clinical variables of the XGBoost model are provided in Supplementary Table S2 and the optimal range of hyperparameters was set with autotune. To mitigate overfitting, regularization techniques (alpha and lambda parameters), and early stopping during training were implemented to prevent fitting noise and memorizing of the training data. Complexity of the model was carefully controlled by tuning the maximum depth of the trees and the number of trees. For graphic output, parameters with a gain ≥ 1% were depicted and the prediction formula to estimate the bleeding risk derived from XGBoost was transformed to a likelihood score and applied to the overall cohort. Thereafter, patients were split into equally sized quartiles according to the corresponding likelihood score. In the Kaplan–Meier transformed survival analyses subgroups were compared with log-rank test. Orthogonal partial least square discriminant analysis (PLS-DA) as well as pattern hunter analysis of associations between chemokine levels and platelet aggregation were performed within “MetaboAnalyst” R package. Correlation matrices were computed with the “corrplot” package and a heatmap of chemokine concentration was created with the “pheatmap” package in R.

## Results

### Baseline characteristics

In the present study, we analysed multiple plasma chemokines/cytokines alongside platelet function derived from a randomized study cohort of patients with CCS undergoing elective PCI [14]. Patients baseline characteristics including pharmaceutical treatment, clinical and laboratory parameters of the study cohort (*n* = 334) are summarized in Table 1 and were reported previously [14]. During the 30-day follow-up period, 21 individuals (6.3%) experienced a relevant bleeding event (BARC 2–5). The median patient age was 67.3 years (IQR 61.5–75.5). Patients with a relevant bleeding event were predominantly female (47.6% versus 22.7%, *p* = 0.010) and shared a significantly lower body mass index (25.1 versus 27.2 kg/comm^2^, *p* = 0.037) as well as reduced haemoglobin levels (13.6 g/dl versus 14.4 g/dl, *p* = 0.019) compared to the individuals who were free from bleeding events (Table 1).

**Table 1:** Baseline characteristics of patient population

	All	BARC < 2	BARC 2–5	*p*-value
	(*n* = 334)	(*n* = 313; 93.7%)	(*n* = 21; 6.3%)	
Female, *n* (%)	81 (24.3)	71 (22.7)	10 (47.6)	**0.010**
Age, years (median, IQR)	67.3 (61.5–75.5)	67.4 (60.7–75)	65 (61.7–77.3)	0.902
Body mass index (median, IQR)	27.1 (24.2–29.8)	27.2 (24.5–29.9)	25.1 (21.8–28.3)	**0.037**
Cardiovascular risk factors and scores on admission				
Arterial hypertension, *n* (%)	296 (88.6)	276 (88.2)	20 (95.2)	0.324
Hyperlipidemia, *n* (%)	298 (89.2)	277 (88.5)	21 (100)	0.100
Diabetes mellitus, *n* (%)	89 (26.7)	82 (26.2)	7 (33.3)	0.474
Current smoking, *n* (%)	67 (20.1)	60 (19.2)	7 (33.3)	0.117
Ex smoking > 6 mo, *n* (%)	78 (23.4)	72 (23)	6 (28.6)	0.559
Atrial fibrillation, *n* (%)	0 (0)	0 (0)	0 (0)	–
Previous CABG, *n* (%)	27 (8.1)	26 (8.3)	1 (4.8)	0.564
Previous MI, *n* (%)	74 (22.2)	69 (22)	5 (23.8)	0.851
Previous stroke, *n* (%)	8 (2.4)	7 (2.2)	1 (4.8)	0.464
Renal function (GFR) (median, IQR)	80 (69.5–92)	80 (69.9–92)	83 (68–95.5)	0.616
PRECISE DAPT Score (median, IQR)	15 (11–21)	15 (11–21)	16 (10–23)	0.945
PARIS Ischemic Score (median, IQR)	2 (2–5)	2 (1–3)	2 (0–3)	0.781
PARIS Bleeding Score (median, IQR)	4 (1–3)	4 (2–4)	5 (3–6)	0.101
ARC-HBR (median, IQR)	0 (0–0)	0 (0–0)	0 (0–0.25)	0.616
Study medication				
Revacept 160 mg, *n* (%)	120 (35.9)	114 (36.4)	6 (28.6)	0.468
Revacept 80 mg, *n* (%)	121 (36.2)	114 (36.4)	7 (33.3)	0.776
Placebo, *n* (%)	93 (27.8)	85 (27.2)	8 (38.1)	0.279
Medication on admission				
ASA, *n* (%)	304 (91)	289 (92.3)	15 (71.4)	**0.001**
Clopidogrel, *n* (%)	179 (53.6)	169 (54)	10 (47.6)	0.571
Statins, *n* (%)	300 (89.8)	280 (89.5)	20 (95.2)	0.396
ACEI/ARB, *n* (%)	255 (76.4)	239 (76.4)	16 (76.2)	0.986
β-blockers, *n* (%)	178 (53.3)	165 (52.7)	13 (61.9)	0.414
Antidiabetic medication, *n* (%)	72 (21.6)	67 (21.4)	5 (23.8)	0.795
Laboratory parameters on admission				
Platelets (10^9^/L) (median, IQR)	223.5 (188.8–259)	224 (188.5–257)	221 (188.5–308.5)	0.559
Hemoglobin (g/dL) (median, IQR)	14.3 (13.4–15.3)	14.4 (13.4–15.3)	13.6 (12.4–14.4)	**0.019**
Leukocytes (10^9^/L) (median, IQR)	6.9 (5.8–8.5)	6.9 (5.8–8.4)	7 (5.5–9.4)	0.815
Neutrophils (%) (median, IQR)	63 (57–68)	63 (57–68)	60.7 (54.5–65)	0.169
Lymphocytes (%) (median, IQR)	25 (20–30)	25 (20–30)	27 (24–34.8)	0.115
Monocytes (%) (median, IQR)	9 (7–10)	9 (7–10)	9 (7.1–11.9)	0.350
Eosinophils (%) (median, IQR)	2 (1–3)	2 (1–3)	2 (1–2.9)	0.399
Basophils (%) (median, IQR)	1 (1–1)	1 (1–1)	1 (0.8–1)	0.591
C-reactive protein (mg/dL) (median, IQR)	0.6 (0.2–1.5)	0.6 (0.2–1.5)	0.6 (0.2–1.1)	0.748
Total cholesterol (mg/mL) (median, IQR)	161 (133.8–200)	161 (133.5–198)	174 (134.5–224.5)	0.377
sGPVI (ng/ml) on admission (median, IQR)	12.2 (8.5–21.1)	12.1 (8.4–20.7)	15.9 (9.6–43.5)	0.132
Platelet functional assays				
COL test on admission (AUC) (median, IQR)	161 (90.5–234)	159.5 (90.8–159.5)	203 (73–264)	0.958
COL test at 48 h (AUC) (median, IQR)	39 (21–81.5)	38 (20–77.5)	75 (36.8–166.5)	0.116
ADP on admission (AUC) (median, IQR)	303 (217.3–479.8)	304 (217.5–484)	238 (199–322)	0.154
ADP test at 48 h (AUC) (median, IQR)	152 (109–199.5)	152 (109–196.5)	150 (82–460)	0.671
30-days follow-up				
MACE (*n*, %)	9 (2.7)	8 (2.6)	1 (4.8)	0.546

*ACE/ARB* angiotensin-converting enzyme inhibitors/angiotensin II receptor antagonists; *ASA* acetylsalicylic acid; *ARC HBR* Academic Research Consortium High Bleeding Risk; *AUC* area under the curve; *BARC* Bleeding Academic Research Consortium; *CABG* coronary artery bypass graft; *IQR* interquartile range; *MACE* major adverse cardiovascular events (including death, myocardial infarction, stroke, or urgent coronary revascularization) *MI* myocardial infarction

Significant (*p* < 0.05) values are highlighted

### Plasma levels of chemokines are associated with ex vivo platelet function

**Fig. 1:**
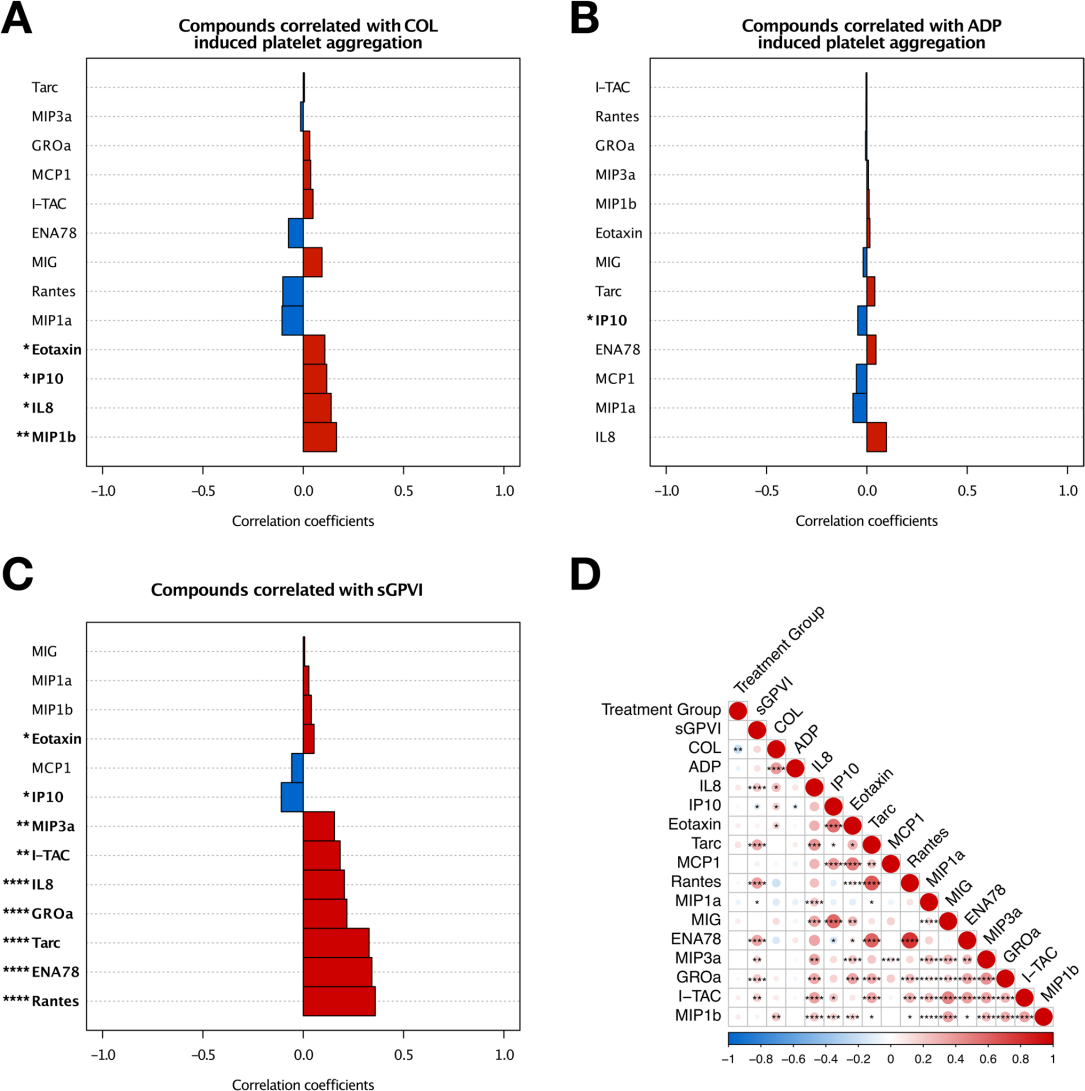
Plasma levels of chemotactic cytokines are associated with a substantial modulation of ex vivo platelet function. **A** Pattern analysis of circulating chemokines (t_48 h_) associated with collagen-mediated platelet aggregation. Bars represent Spearman correlation coefficients and are coloured according to the direction of the relationship. Asterisks represent significant (*p* < 0.05) correlations. **B** Correlation analysis of adenosine diphosphate (ADP) induced platelet aggregation and plasma chemokine concentration at t_48 h_. **C** sGPVI concentration in patients with CAD is associated with a shift in the chemokine profile (t_48 h_). Compounds are highlighted according to the referring correlation coefficient. **D** Correlation matrix synopsizing correlations of chemokines (t_48 h_), study drug treatment, and sGPVI concentrations alongside ex vivo platelet functional assays. Spearman’s ρ is coloured and dots are plotted proportional to correlation coefficients. *p < 0.05, **p < 0.01, ***p < 0.001, ****p < 0.0001

We performed a comprehensive correlation analysis to elucidate the interplay between distinct platelet-derived chemokines (t_48 h_) and platelet reactivity (Fig. 1). Collagen-induced platelet aggregation was significantly (*p* < 0.05) associated with high levels of circulating eotaxin (C–C motif chemokine ligand 11 [CCL11]), IP10 (interferon gamma-induced protein 10, C-X-C motif chemokine ligand 10 [CXCL10]), IL8 (interleukin 8, C-X-C motif chemokine ligand 8 [CXCL8]), and MIP1b (macrophage inflammatory protein 1b, C–C motif chemokine ligand 4 [CCL4]) (Fig. 1A). In contrast, ADP-mediated platelet aggregation was less dependent on circulating chemokines, but only high concentrations of IP10 were significantly (*p* < 0.05) associated with platelet hyperreactivity (Fig. 1B). In line with collagen-induced platelet aggregation, we assessed the association between the soluble collagen receptor GPVI (sGPVI) and circulating chemokines/cytokines (Fig. 1C). We found that sGPVI concentration significantly (*p* < 0.05) correlated with plasmatic concentrations of eotaxin, MIP3α/CCL20, I-TAC/CXCL11, IL8, GROα/CXCL1, Tarc/CCL17, ENA78/CXCL5, and Rantes/CCL5. Contrarily, IP10 was inversely associated (*p* < 0.05) with sGPVI (Fig. 1C). Here, it was striking that correlation was strongest between chemokine expression and sGPVI levels indicating a major impact of the mediators on collagen-dependent platelet aggregation. Likewise, extensive correlation analyses unveiled a compelling interrelationship between distinct plasma chemokines (Fig. 1D). It was further noticeable that both conventional platelet function tests at baseline and after administration of study medication did not unveil significant differences between patients with relevant bleeding and those without adverse events (Supplementary Fig. S2).

### Plasma levels of eotaxin are associated with bleeding events in patients with CAD

**Fig. 2:**
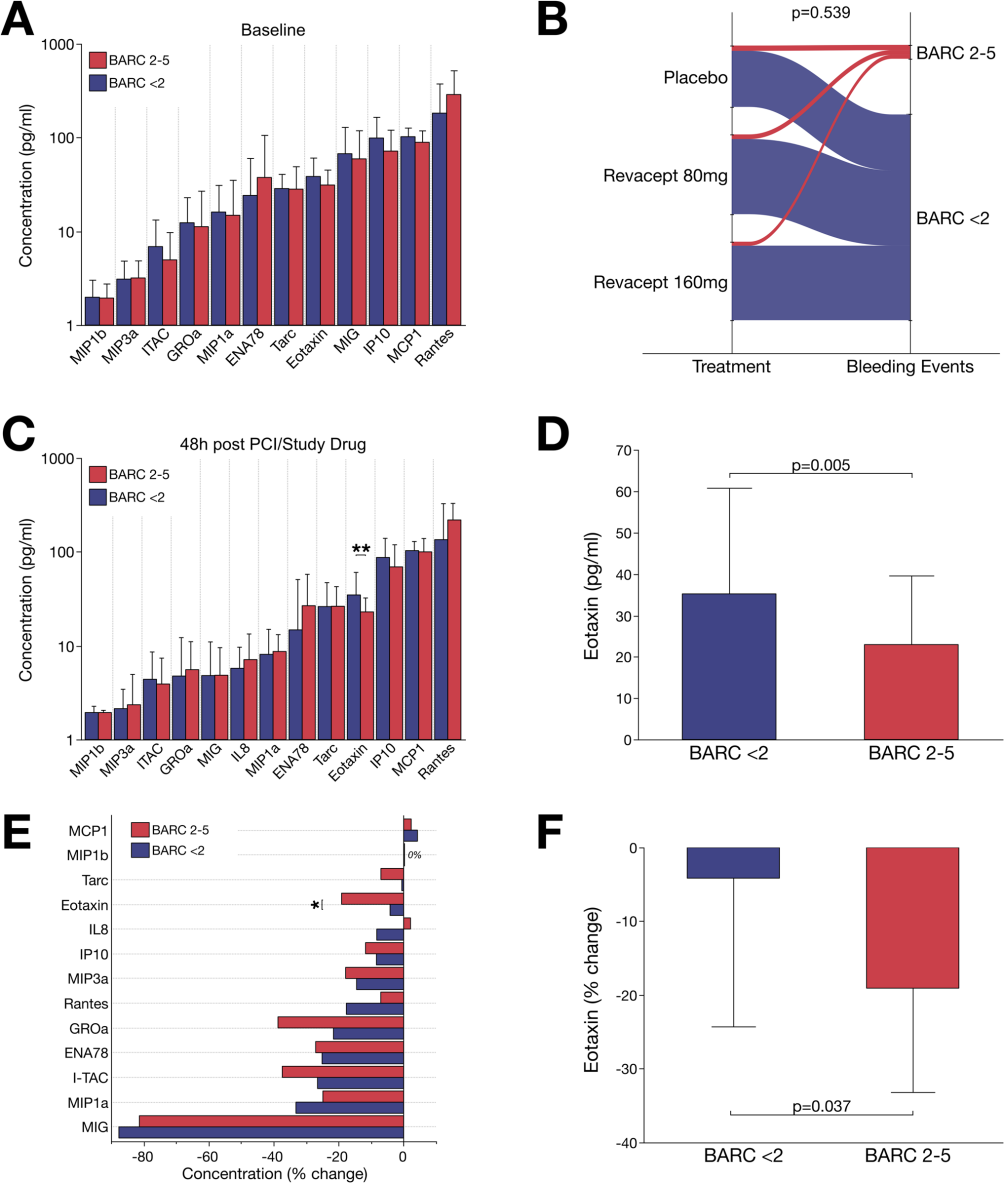
CAD patients with adverse bleeding events exhibit a substantial deficiency of chemotactic eotaxin. **A** Bar chart subsumes comparison of chemokines between patients with relevant bleeding to those without adverse events at baseline (t_0 h_). There were no observations of an altered chemokine signature prior PCI and administration of antiplatelet therapy or study drug treatment. **B** Sankey plot depicting proportions of adverse bleeding events between individual study drug treatment groups. Thus, treatment with GPVI antagonist revacept was not associated with bleeding incidences during the follow-up period. **C** In the overall cohort bar charts compare chemokine concentrations after PCI and allocation of study drug (t_48 h_) in line of bleeding events. *p < 0.05. **D** Postinterventional eotaxin concentration (t_48 h_) was significantly (*p* < 0.05) reduced in patients with relevant bleeding (BARC 2–5) compared to patients without adverse events regardless of whether they received revacept or placebo **E** Bar chart exhibiting the percentage change of distinct chemokines before and after PCI and study drug administration. *p < 0.05. **F** Plasmatic eotaxin is critically (*p* < 0.05) decreased in patients with adverse bleeding events after PCI and antiplatelet treatment

In the analysis of relevant clinical events, we found that chemokine concentrations prior to PCI and allocation of revacept or placebo did not differ between patients with major bleeding and those without bleeding events (Fig. 2A). Further, application of study medication including revacept was not associated (*p* = 0.539) with relevant bleeding (Fig. 2B).

However, in the overall cohort post-interventional concentration of eotaxin was significantly (*p* = 0.005) reduced when comparing patients with adverse bleeding events to individuals free from relevant bleeding (Fig. 2C and D). In addition, the post-interventional drop in eotaxin concentration from baseline levels was most prominent (*p* = 0.037) in patients with adverse bleeding compared to patients without such events (Fig. 2E and F). Further, bleeding severity assessed by BARC was significantly but modestly associated with postinterventional eotaxin concentration (*r* = − 0.153, *p* = 0.006) as well as change of eotaxin from baseline (*r* = − 0.114, *p* = 0.039) (Supplementary Fig. S3). Additionally, eotaxin was inversely associated with C-reactive protein indicating an interrelation to systemic inflammation (Supplementary Fig. 4). To test whether systemic conditions influence the expression of platelet-derived chemokines, we performed partial least square discriminant analysis (PLS-DA). The multivariate models including diabetes mellitus, dyslipidaemia, obesity, smoking status, arterial hypertension, and impaired renal function unveiled a minor impact of the risk factors on the chemokine signature (Supplementary Fig. S5). Postinterventional eotaxin concentration between patients with and without cardiovascular risk factors did not vary significantly (Supplementary Fig. S6) and after adjustment for age, gender, BMI, renal function, and haemoglobin, post-interventional eotaxin concentration was shown to be independently associated with BARC 2–5 bleeding in a multivariable regression analysis (Supplementary Table S3). Interestingly, eotaxin alone exhibited a superior prediction accuracy in assessing the endpoint of BARC 2–5 when compared to sGPVI concentrations or validated bleeding risk scores. Thus, AUC in univariable regression analysis was highest for eotaxin (ROC AUC = 0.682) when compared PRECISE DAPT (ROC AUC = 0.496), ARC-HBR (ROC AUC = 0.523), or PARIS bleeding risk (ROC AUC = 0.606) in this study (Supplementary Fig. S7).

### Revacept treatment is associated with changes in post-interventional chemokine plasma levels

**Fig. 3:**
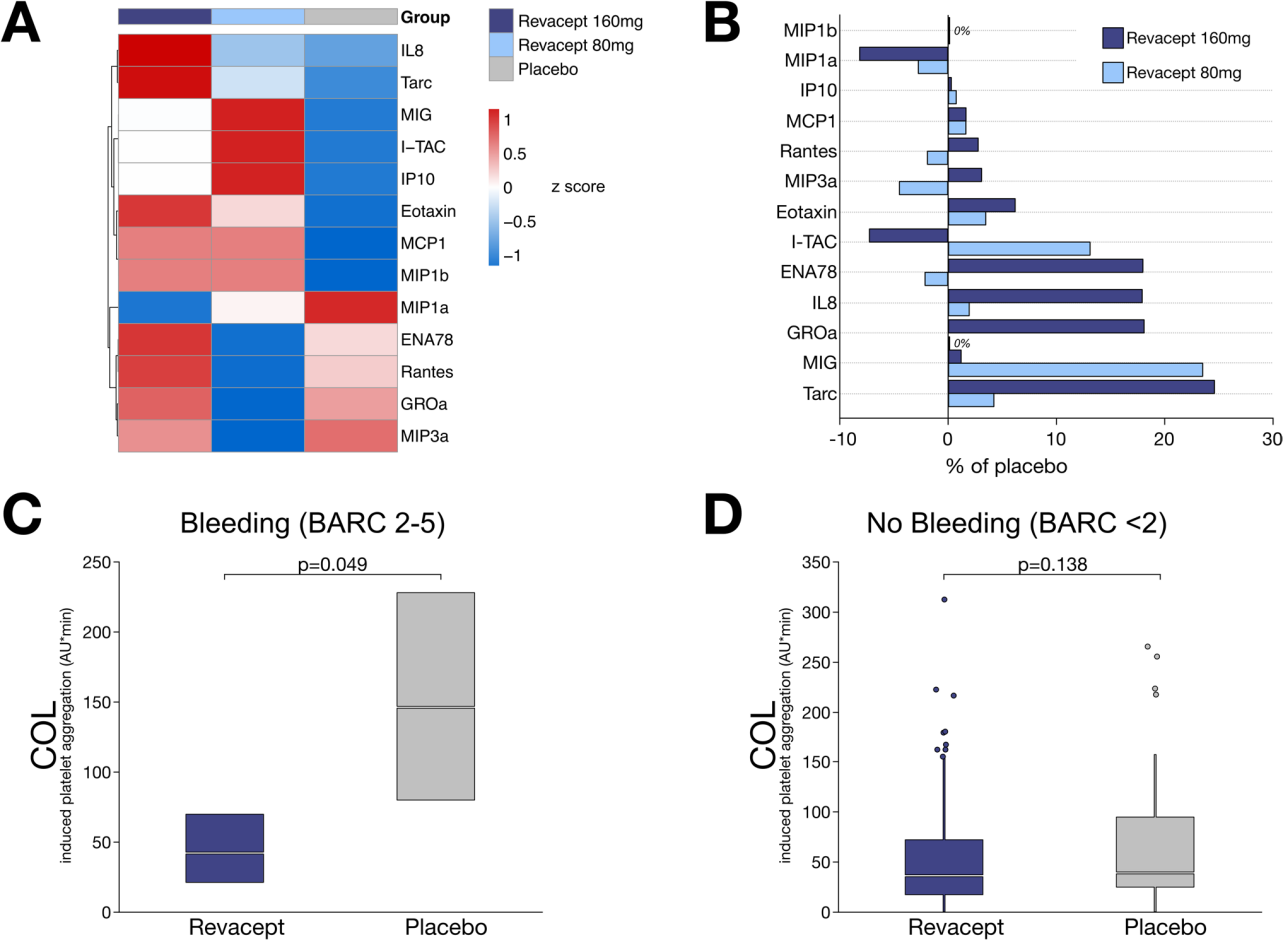
The plasma chemokine profile is influenced by the antiplatelet treatment with revacept in patients with coronary artery disease. **A** Hierarchical clustering analysis of the analysed chemokines (t_48 h_) according to the referring treatment group. Z-scores of median concentrations were coloured according to up-regulation (red) or down-regulation (blue) of distinct mediators. **B** Bar chart showing the dose-dependent regulation of chemokines (t_48 h_) in revacept-treated patients in contrast to placebo. None of the chemokines differed significantly (*p* < 0.05) between treatment groups. **C** Collagen-dependent platelet aggregation was significantly (*p* < 0.05) decreased in patients with relevant bleeding after administration of Revacept when compared to patients receiving placebo (t_48 h_). **D** Ex vivo collagen-dependent platelet aggregation did not differ between treatment subgroups in patients without adverse events (t_48 h_)

In the present placebo-controlled study, we prospectively analysed the chemokine profile of patients receiving revacept in addition to conventional DAPT. Here, we found that the chemokine profile of patients allocated to receive revacept was divergent compared to those patients receiving placebo (Fig. 3A). Moreover, comparison of circulating chemokines elucidated a dose-dependent effect of revacept treatment on individual chemotactic cytokines. Of note, most mediators, including eotaxin and MIG, were up-regulated by trend in patients receiving revacept but not to a statistically significant level (Fig. 3B). Interestingly, in patients with adverse bleeding events receiving revacept treatment (80 mg/160 mg) we found that platelet function was substantially (*p* = 0.049) reduced in contrast to those individuals with adverse bleeding receiving placebo (Fig. 3C). However, this observation was inapparent in individuals without adverse bleeding events, as collagen-mediated platelet aggregation was equivalent (*p* = 0.138) in revacept-treated patients when compared to those patients who received placebo (Fig. 3D).

### Plasma levels of chemokines improve stratification of bleeding risks

**Fig. 4:**
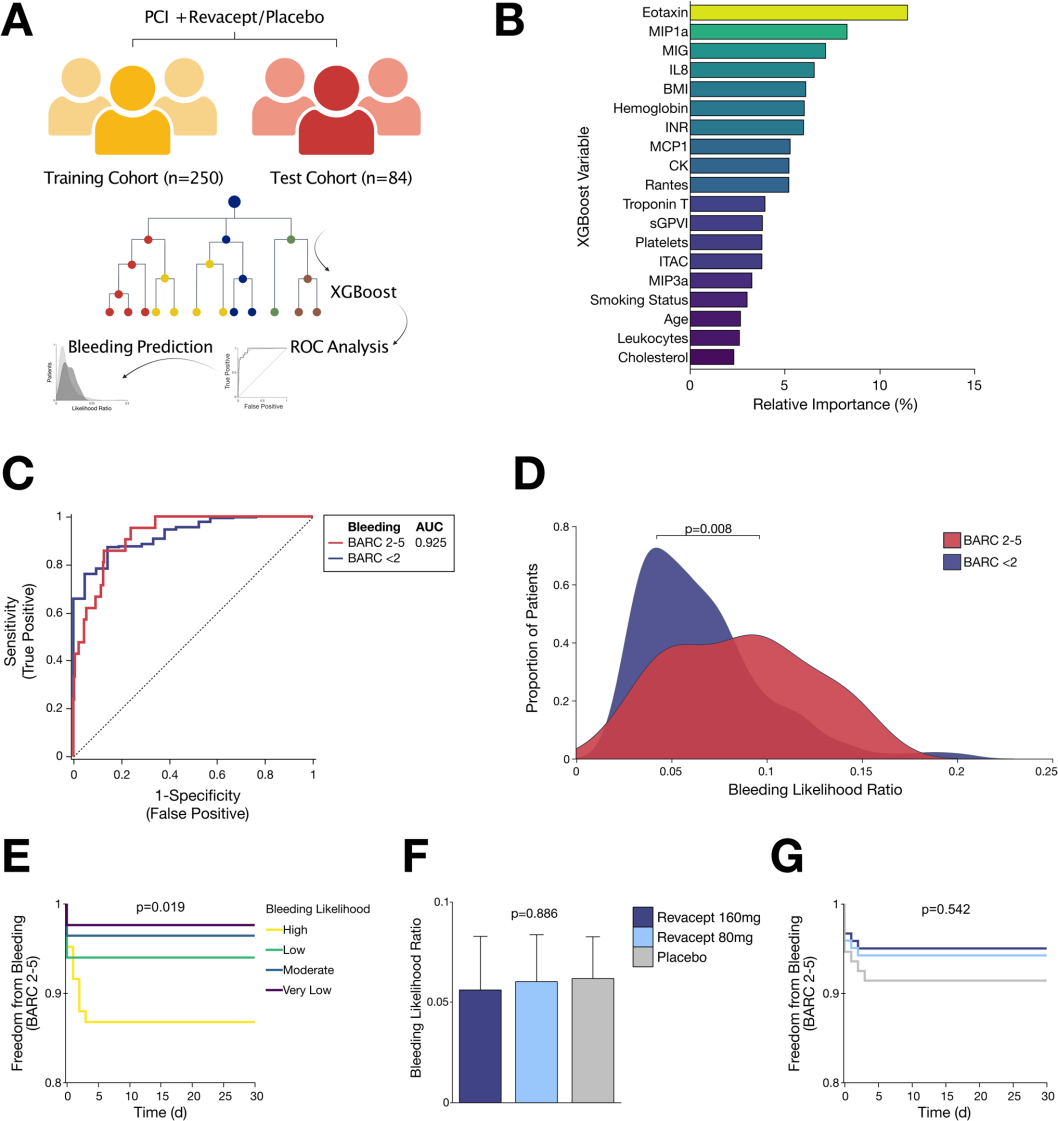
Machine learning identifies a chemokine signature in CAD associated with adverse bleeding events after PCI and antiplatelet therapy. **A** Methodological outline of the risk estimation employing XGBoost models of risk parameters including plasma chemokines (t_48 h_) in CAD patients of this study. **B** Most important variables predicting adverse bleeding events based on a machine learning algorithm (XGBoost). Included parameters are sorted according to relative importance of gain parameters and mostly comprise plasma chemokine levels. **C** Receiver operating characteristic (ROC) curves depicting the diagnostic accuracy of XGBoost model of the training cohort. Area under the curve (AUC = 0.93) of the cross-validated machine learning algorithm yields a high degree of accuracy in identifying patients with bleeding events. **D** Transformed likelihood formula of bleeding risk from XGBoost model was plotted against actual bleeding events. A substantial (*p* < 0.05) separation of patients with relevant bleeding from those without adverse bleeding demonstrates the accuracy of risk assessment integrating plasma chemokine levels. **E** Kaplan–Meier curves subsuming quartiles according to the individual bleeding likelihood score. Patients at increased bleeding risk based on plasma chemokine levels are significantly (*p* < 0.05) more likely to suffer from future haemorrhagic events. **F** The individual bleeding likelihood from XGBoost model appears to differ little (*p* = 0.9) between treatment subgroups. **G** Kaplan–Meier curves of freedom from bleeding events based on treatment subgroups exhibits an insignificant (*p* = 0.5) dose-dependent reduction of bleeding events in revacept-treated patients in contrast to placebo

To estimate the individual hazard of relevant bleeding in CCS patients undergoing PCI, all patients enrolled into this prospective study were randomly categorized into a training cohort and a test cohort (Fig. 4A). In both subgroups, we performed XGBoost modelling integrating important clinical risk parameters including circulating chemokine concentrations (Supplementary Table S2). In the machine learning algorithm assessing the postinterventional bleeding risk in CCS patients undergoing PCI, the most influential variables included mainly chemokine concentrations post-PCI. Here, it was noticeable that chemokines including eotaxin, MIP1α, and MIG were the most important estimators of an increased bleeding risk (Fig. 4B). Likewise, sGPVI showed an influential importance in stratification of adverse bleeding events, and thus modulation of collagen-depended platelet aggregation might play an important role of coagulation homeostasis in CAD patients (Fig. 4B). The final XGBoost model unveiled a robust accuracy with an overall accuracy of 94% in both, training, and test cohort (AUC = 0.93 in the training cohort, AUC = 0.67 in the test cohort) (Fig. 4C). The model output was then applied to the overall cohort (Supplementary Fig. S8). Based on predicted likelihood for adverse bleeding events, the machine learning model showed a robust accuracy of both patients with relevant bleeding and those without adverse events. Thus, the bleeding likelihood score of clinical parameters and chemokine concentrations in patients with adverse bleeding was substantially (*p* = 0.008) increased in contrast to patients without bleeding events (Fig. 4D). According to predicted bleeding likelihood, individuals were divided into quartiles and Kaplan–Meier analysis uncovered a significant (*p* = 0.019) segregation of likelihood quartiles with regard to actual bleeding events (Fig. 4E). However, bleeding likelihood score did not differ significantly among the treatment subgroups (*p* = 0.886), but the individual bleeding hazard was reduced by trend in revacept-treated patients when compared to those receiving placebo (Fig. 4F). Similarly, Kaplan–Meier estimator showed non-significant (*p* = 0.542) dose-dependent reduction of the bleeding risk in revacept-treated patients compared to patients receiving placebo (Fig. 4G).

## Discussion

The major findings of the present study are: (1) Plasma levels of chemokines/cytokines are associated with ex vivo platelet aggregation in patients with CCS undergoing PCI. (2) Plasma levels of eotaxin (CCL11) are associated with bleeding events in patients with CAD. (3) Administration of the collagen antagonist revacept is associated with changes in postinterventional chemokine plasma levels. (4) Plasma levels of chemokines are associated with an improved assessment of individual bleeding risks.

Thus, our findings suggest that assessment of distinct chemokines may improve the diagnostic value for evaluation of the bleeding risk and might offer novel diagnostic opportunities to individualize the duration of DAPT in CCS patients treated with PCI.

Platelet activation and hyper- or hyporesponsiveness are associated with thrombo-ischemic events and bleeding in patients undergoing PCI [16]. On-treatment ex vivo platelet hyperresponsiveness predicts the occurrence of ischemic events and stent thrombosis after PCI [17, 18]. In turn, enhanced ex vivo platelet hyporesponsiveness is associated with bleeding events [19]. Platelet activation and degranulation results in a substantial release of a variety of chemokines including CXCL1, PF4 (CXCL4), CXCL5, CXCL7, IL-8, CXCL12, MIP-1α, eotaxin (CCL11), RANTES (CCL5) and others [1]. Release of platelet chemokines trigger inflammation via interaction of leukocytes or monocytes (thrombo-inflammation) [20]. In turn, distinct chemokines have pro- and anti-activating influence on platelets [6, 7]. Systemic inflammation is associated with bleeding diathesis in patients with CAD [21]. Further, inflammation is associated with platelet hyperreactivity and thromboischemic events [22]. Therefore, we hypothesized that systemic levels of plasma chemokines may have an impact on bleeding events in patients undergoing PCI and treatment with combined antiplatelet therapy.

Our observation of an association between distinct chemokines and ex vivo collagen-induced platelet aggregation as well as levels of liberation of soluble GPVI (sGPVI) and inflammatory biomarkers such as CRP implies that the systemic inflammation modulates the function of circulating platelets. We found that among the tested chemokines, none of the inflammatory mediators determined prior to PCI was associated with bleeding events. However, levels of eotaxin significantly dropped after PCI. The change of eotaxin levels pre- and post-PCI was significantly associated with the occurrence and severity of bleeding, albeit correlation strength was modest and number of patients with severe bleeding was limited. Eotaxin is highly expressed in atherosclerotic plaques and critical for neovascularization and coagulation including thrombus formation [20, 23]. Further, eotaxin concentrations correlate with the extent and number of relevant coronary stenosis and promote atheroprogression in patients with CAD [24, 25].

Following PCI, the significant reduction in coronary stenosis and the resulting depletion of eotaxin deposits may provide a basis for hypothesizing a decrease in angiogenic and prothrombotic chemokine levels. Previously, a drop of CCL26, a member of the eotaxin family, was linked to adverse cardiovascular events in patients with CAD [26].

Sufficient reperfusion of the myocardium critically impacts thrombo-inflammation, and compensatory pathways may interrelate with signalling cascades including eotaxin [27, 28]. On the contrary, a disequilibrium and critical down-regulation of eotaxins might result in adverse effects and thus result in bleeding events.

In this study, concentrations of chemokines including eotaxin did not differ prior to PCI and were robust to coexisting risk factors. Although we cannot provide direct evidence, the down-regulation of eotaxin and its association with platelet reactivity could be a pathophysiological link to bleeding in CCS patients. Therefore, we hypothesized that patients with low eotaxin concentrations might take advantage of intensified clinical monitoring and might benefit from short-term dual antiplatelet treatment following PCI to prevent major bleeding events. However, validation of the results is mandatory to define thresholds and investigate long-term outcomes. Assessment of chemokines such as eotaxin might contribute to individual patient care after PCI since guiding DAPT according to platelet functional analysis failed to improve long-term outcomes after coronary stenting [29].

Interestingly, the administration of the collagen antagonist revacept shows a dose-dependent effect on plasma chemokine levels. However, a significant correlation was observed only when considering the total sGPVI concentration, including endogenous levels. Among others, eotaxin was prominently affected compared to placebo controls but failed to reach significance, which might hint at previous findings that revacept did not reduce the bleeding risk after PCI [14]. Changes of chemokines in revacept-treated patients, including MIG, an important anti-atherosclerotic mediator and opponent of eotaxin, might reflect a double role to prevent atheroprogression and to maintain hemostasis [30].

In our machine learning model assessing the postinterventional bleeding risk in CCS patients undergoing PCI, the most influential factors, among other demographic and laboratory parameters, included mainly chemokines and most prominently—eotaxin. A substantial separation of patients with relevant bleeding from those without adverse bleeding underscores the accuracy of risk prediction integrating plasma chemokine levels.

## Limitations

We are fully aware that the strength of our conclusion that systemic chemokine signatures are associated with bleeding events in PCI patients has obvious limitations. The number of adverse events in this study was limited, and reliable clinical scores indicated a low to moderate risk of developing ischemic and bleeding events, as the study primarily evaluated the feasibility and safety of revacept in addition to conventional DAPT. Additionally, the number of thromboischemic and major bleeding events in this cohort of CCS patients post-PCI was low, and the study does not allow to draw conclusions regarding the type or location of bleeding. The findings may have differed substantially in an ACS cohort with an increased rate of major cardiovascular events and, especially, bleeding due to high-potency antiplatelet therapy. Thus far, the significant results are limited to this low-risk cohort. Thus, the generalizability of this study may be confined by the limited prevalence of adverse events and a larger prognostic study is warranted to contextualize these interesting results with further research. Prediction accuracy of machine learning including chemokine concentrations might be restricted by model and cohort-specific influences. Thus, external validation of the novel results would increase the reliability and enhance the significance of an aberrant chemokine expression associated with adverse bleeding. In line with this, we are fully aware that a pre-specified set of chemokines does not encompass the full scope of thrombo-inflammation and coagulation and may thus affect the interpretation of this study. To screen for chemokines associated with bleeding, translational metabolomic assays (e.g., mass spectrometry) could provide additional information. Further, chemokine concentrations might vary with coexisting conditions including disease severity and comedication. However, multivariate analysis demonstrated a minor influence of cardiovascular risk factors and concomitant diseases on the chemokine profile that is critically altered in patients with adverse bleeding events. In addition, the cohort comprised exclusively of patients with stable CAD, and unveiled that chemokine concentrations and platelet function did not alter between patients with adverse events before treatment. However, acute processes, including plaque rupture and inflammatory responses, need to be taken into account as they might impact the chemokine signature. 

Ultimately, we are convinced that our data, as presented, provides an interesting hypothetical framework for including parameters of systemic inflammation and platelet markers in the stratification of bleeding events. The identification of an individual risk for bleeding may assist to tailor the duration and strength of antiplatelet therapy.

## Supplementary Information

Below is the link to the electronic supplementary material.

Supplementary file1 (DOCX 1130 KB)

## Data Availability

The data that support the findings of this study are available on request from the corresponding author.
