# Supplementary material for: Plasma chemokines indicate enhanced bleeding in patients with chronic coronary syndrome undergoing percutaneous coronary stenting
Source: Clin Res Cardiol. 2025 May 21;114(8):1071–83. doi: 10.1007/s00392-025-02675-8 (PMC12283909; doi:10.1007/s00392-025-02675-8)
Supplement: Supplementary file 1 — Supplementary file1 (DOCX 1130 KB) [file 392_2025_2675_MOESM1_ESM.docx]

**Plasma chemokines indicate enhanced bleeding in patients with chronic coronary syndrome undergoing percutaneous coronary stenting**

**Short title:** Bleeding and chemokine profile

Tobias Harm^1^ MD, Shqipdona Lahu^2^ MD, Katharina Mayer^2^ MD, Dominik Rath^1^ MD, Tobias Geisler^1^ MD, Karin Anne Lydia Müller^1^ MD, Marion Janisch^2^ MD, Kristin Adler^5^ MD, Götz Münch^5^ MD, Steffen Massberg^3,4^ MD, Adnan Kastrati^2,3^ MD, Meinrad Paul Gawaz^1,*^ MD

^1^Department of Cardiology and Angiology, University Hospital Tübingen, Eberhard Karls University Tübingen, Germany

^2^Deutsches Herzzentrum München, Klinik für Herz- und Kreislauferkrankungen, Technische Universität, Munich, Germany

^3^German Center for Cardiovascular Research (DZHK), Partner Site Munich Heart Alliance, Munich, Germany

^4^Department of Cardiology, Munich University Clinic, Ludwig-Maximilian University of Munich, Munich, Germany

^5^AdvanceCOR GmbH, Martinsried, Germany

***Correspondence to:**

**Meinrad Paul Gawaz**

Department of Cardiology and Angiology

University Hospital Tübingen, Eberhard Karls University Tübingen

Otfried-Müller-Str. 10, 72076 Tübingen, Germany

Tel.: +49 (0) 7071 29 83688

Fax: +49 (0) 7071 29 5749

E-Mail: meinrad.gawaz@med.uni-tuebingen.de

**Supplementary Methods**

**Study Design**

The study was a multicentric phase II, placebo-controlled trial. Therefore, patients with chronic coronary syndrome (CCS) were enrolled from 9 centers in Germany from November 20, 2017, to February 27, 2020. The study adhered to the Declaration of Helsinki, and its protocol received approval from the ethics committee at each participating site (Ethics committee Berlin Land, 18/0172-EK15; Ethics committee Rheinland-Pfalz, 837.222.17 (11056); University Frankfurt, 229/17B; University München LMU, 746-16mb; University Regensburg, 16-363-113; University Tübingen 410/2018AMG2). The 30-days clinical follow up follow-up period ended on March 27, 2020. Platelet function was assessed prior to randomization of patients and double-blinded allocation of Revacept or placebo, respectively, as well as 48 hours after PCI and antiplatelet treatment. After hospital discharge, a close-meshed clinical follow-up period over thirty days was performed to screen for adverse cardiovascular events including a composite of death and myocardial injury as well as bleeding events. The latter was defined according to Bleeding Academic Research Consortium (BARC) and relevant bleeding events were classified by BARC type 2-5, whereas patients with no or mild bleeding were summarized by BARC ≤1. Process-related access site bleeding occurred in 36 patients; 4 patients experienced spontaneous extremity bleeding, 4 had nosebleeds, 6 had skin bleeding, 3 had urogenital bleeding, and 1 patient had ventricular bleeding.

All patients completed a follow-up and were included for further analysis.

**Supplementary Tables & Figures**

**Supplementary Table S1.** Inclusion and exclusion criteria

| **Inclusion Criteria** |  |
| --- | --- |
| Signed written informed consent | Stable CAD |
| Patients >18 years of age | Angiographic confirmation of CAD & indication for PCI |
|  |  |
| **Exclusion Criteria** |  |
| WOCBP unwilling/unable to use contraception for 4 weeks after study drug administration | Women who are pregnant or breastfeeding or are planning pregnancy |
| Women with a positive pregnancy test on enrolment | Elevated high sensitivity cardiac troponin T levels on admission |
| Patients receiving Prasugrel or Ticagrelor within 7 days prior to randomization | History of hypersensitivity, contraindication or adverse reaction to the study drug |
| History of bleeding diathesis or active bleeding within the last 30 days | Intracerebral hemorrhage or trauma within the last 3 months |
| Thrombocytopenia (platelet count <30000/mm^3^) on admission | Sustained hypertension (BP > 179/109 mmHg) on admission |
| Renal failure (glomerular filtration rate< 30ml/min and/or dialysis) | Severe systemic disease with life expectancy < 1 year |
| Unable to provide informed consent (e.g. severe dementia, or psychosis) | Suspected poor capability to follow instructions and cooperate |
| Patients with an indication for anticoagulant therapy | Participation in any other clinical interventional trial within less than 30 days |
| Any other contraindication to perform PCI | Any planned additional PCI or surgery within 30 days after randomization |
| Transaminase level > 5-fold the upper normal range limit | Prisoners or subjects who are involuntarily incarcerated |
| Subjects under compulsory detention for psychiatric or physical illness |  |
|  |  |
| *BP, blood pressure; CAD, coronary artery disease; PCI, percutaneous coronary intervention; WOBC, women of childbearing potential.* | |

**Supplementary Table S2.** Variables integrated in the XGBoost machine learning model

| **Variable** | **Variable** |
| --- | --- |
| Study Medication (Revacept/Placebo) | IL8 (C_48h_ + % change) |
| Sex Category | IP10 (C_48h_ + % change) |
| Age | Eotaxin (C_48h_ + % change) |
| Arterial Hypertension | Tarc (C_48h_ + % change) |
| Dyslipidemia | MCP1 (C_48h_ + % change) |
| Smoking Status | Rantes (C_48h_ + % change) |
| Diabetes Mellitus | MIP1a (C_48h_ + % change) |
| Renal Function (GFR) | MIG (C_48h_ + % change) |
| Hemoglobin | ENA78 (C_48h_ + % change) |
| Platelets | MIP3a (C_48h_ + % change) |
| Leukocytes | GROa (C_48h_ + % change) |
| INR | I-TAC (C_48h_ + % change) |
| Creatine Kinase | MIP1b (C_48h_ + % change) |
| C-reactive Protein |  |
| Total Cholesterol |  |
| Troponin T |  |
| Radial/Femoral Access |  |
| BMI |  |
| sGPVI (C_0h_ + C_48h_) |  |
| ADP-induced Platelet Aggregation (C_48h_ + % change) | |
| COL-induced Platelet Aggregation (C_48h_ + % change) | |

**Supplementary Table S3.** Nominal logistic model identifies post-interventional eotaxin concentrations as independently associated with BARC 2–5 bleeding.

| **Multivariable Regression Analysis (p=0.047)** | | | | |
| --- | --- | --- | --- | --- |
| **Variable** | **ß** | **Std. Error** | **p-Value** |  |
| Eotaxin (_t48h_) | -0.03 | 0.01 | **0.042** |  |
| Age | -0.01 | 0.02 | 0.162 |  |
| Female | 0.75 | 0.53 | 0.161 |  |
| BMI | -0.11 | 0.06 | 0.061 |  |
| Renal Function (GFR) | -0.06 | 0.55 | 0.248 |  |
| Hemoglobin | -0.23 | 0.19 | 0.211 |  |
|  |  |  |  |  |
| *BMI, body mass index; GFR, glomerular filtration rate; Std., standard.* | | | |  |

******

**Supplementary Figure S1. Screening, randomization, treatment, follow-up, and analysis of patients with chronic coronary syndrome enrolled into this prospective study.** Evaluation of study objects was performed carefully from randomization until death, withdrawal of consent, or the last contact date as indicated in the initial study report of the “*Lesion Platelet Adhesion as Selective Target of Endovenous Revacept in Patients with Chronic Coronary Syndrome*” study (ISAR-PLASTER; Rev/CAD/02 EudraCT Number: 2015-000686-32).

**Supplementary Figure S2.** Comparison of **(A)** collagen-induced platelet aggregation and **(B)** ADP-induced platelet aggregation at baseline, prior administration of study medication did not vary between patients with relevant bleeding events and those without adverse events. Likewise, **(C)** collagen-induced platelet aggregation and **(D)** ADP-induced platelet aggregation did not reveal significant differences between patient subgroups 48 h after administration of study drugs.

**
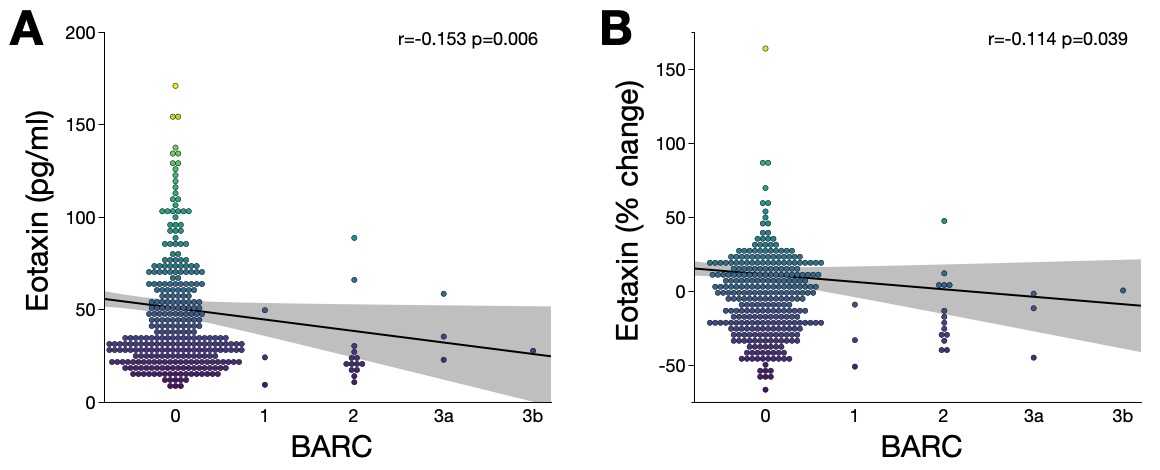
Supplementary Figure S3.** Spearman correlation analyses assessing the association of bleeding severity by BARC (Bleeding Academic Research Consortium) score and **(A)** postinterventional eotaxin concentrations as well as **(B)** relative change of eotaxin concentrations.


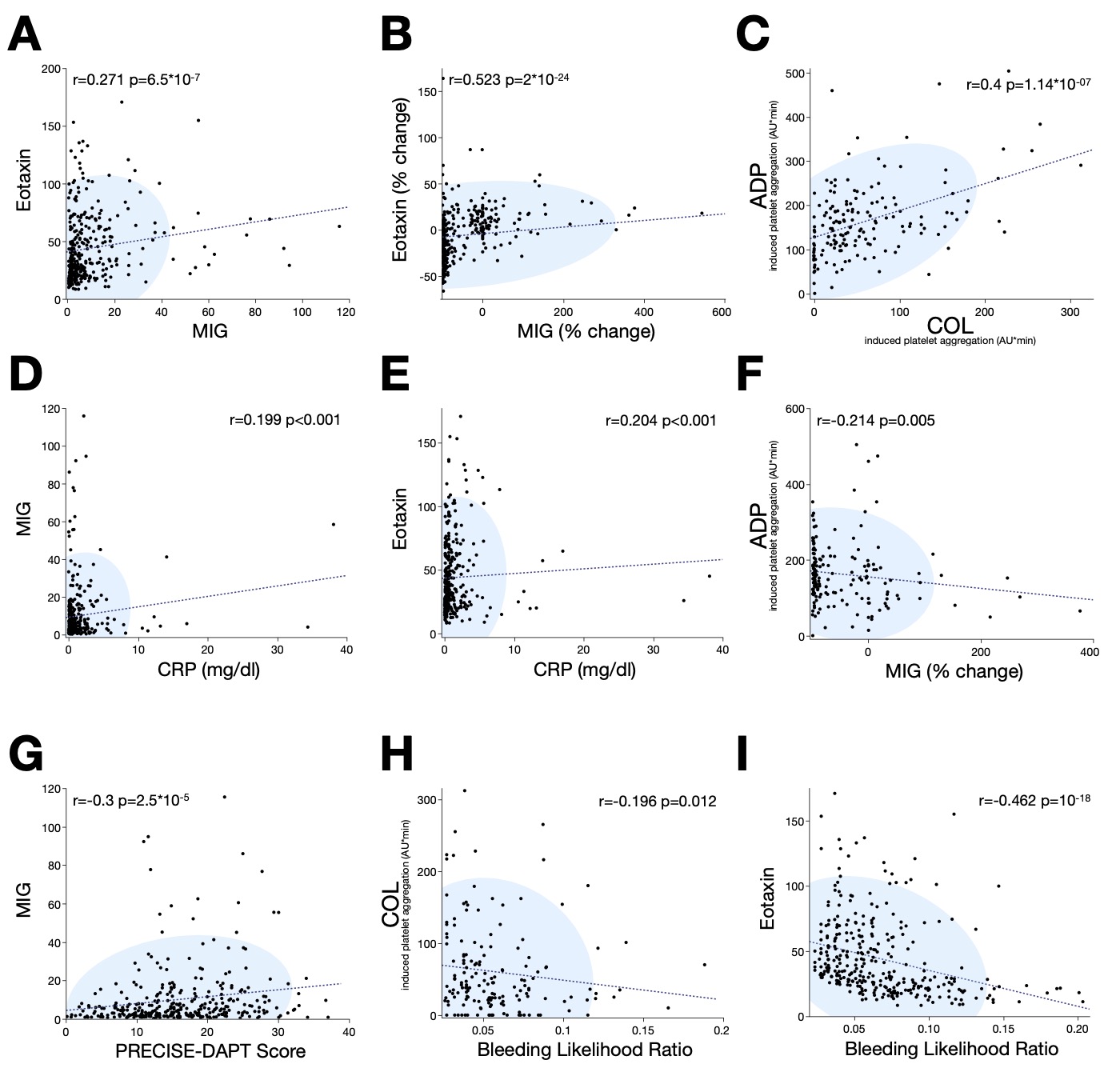
**Supplementary Figure S4. Plasma chemokines correlate with clinical parameters in patients with CAD.** **(A-I)** Comprehensive correlation analysis including important chemokines (i.e. Eotaxin, MIG), inflammatory markers (C-reactive protein), platelet functional data, and bleeding risk estimators (PRECISE-DAPT Score, Bleeding Likelihood Ratio of XGBoost Model). Pearson correlation coefficients and corresponding and significant p-values (<0.05) are highlighted.

**
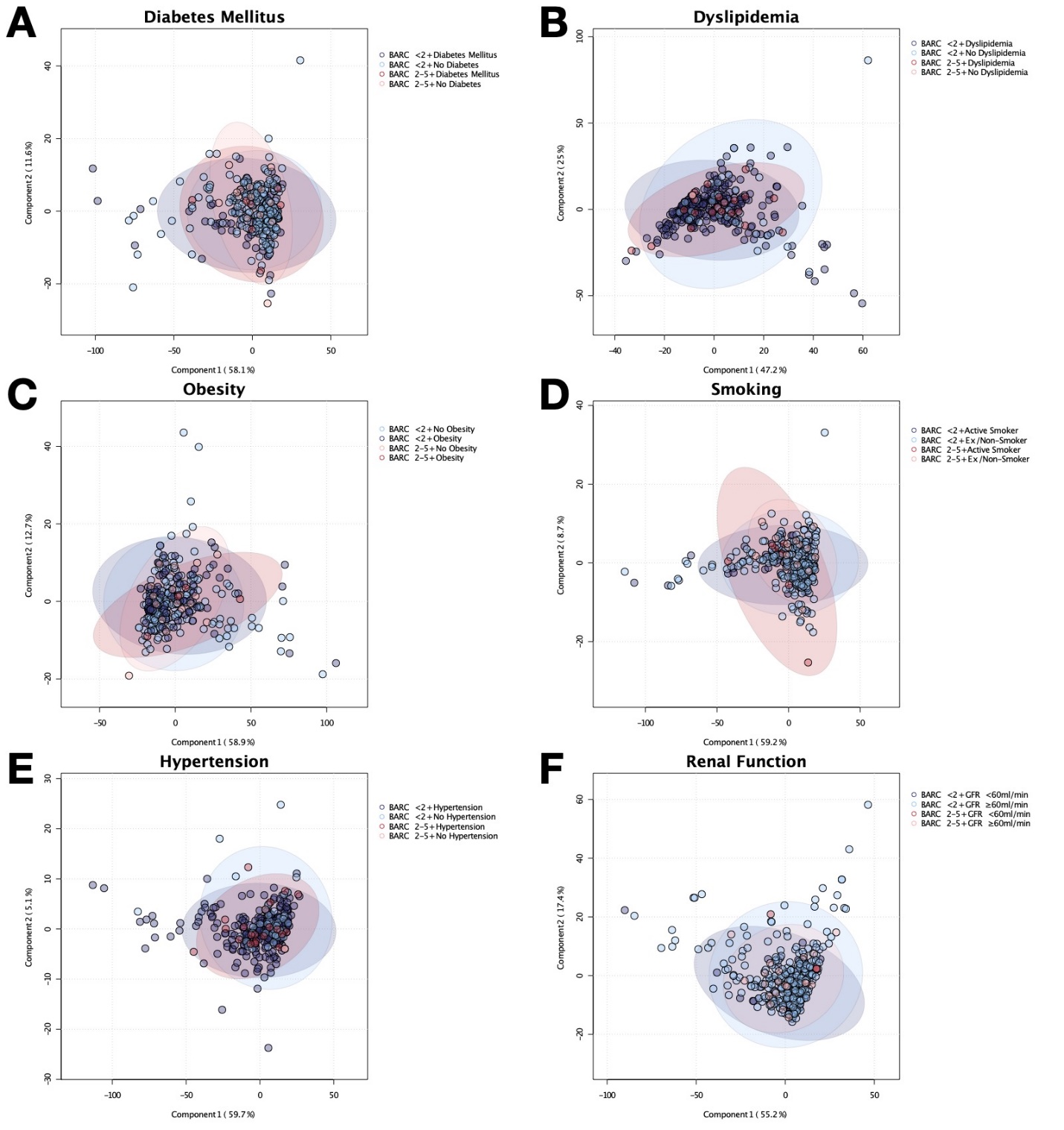
Supplementary Figure** **S5.** **Effect of cardiovascular risk factors on the chemokine profile. (A-F)** Orthogonal partial least squares discriminant analysis (PLS-DA) assessing the impact of diabetes mellitus, dyslipidemia, obesity, smoking status, arterial hypertension, and impaired renal function on platelet-derived chemokine species. Data were based on normalized chemokine concentrations in all patients enrolled into this study (n=334). The subcohorts are colored and labelled, accordingly and a homogenous spreading of the patient subgroups indicates a minor influence of assessed risk factors and diseases on the chemokine signature in this study.

**Supplementary Figure** **S6.** **Juxtaposition of postinterventional eotaxin levels between patients with cardiovascular risk factors and those without coexisting conditions.** Concentrations of the chemokine found to be up-regulated in patients with adverse bleeding did not differ significantly (p<0.05) between individual risk groups including obesity, diabetes mellitus, dyslipidemia, arterial hypertension, impaired renal function, and smoking.

**Supplemental Figure S7.** ROC Analysis of univariable regression analysis assessing the risk for BARC 2-5 bleeding by integration of eotaxin or sGPVI concentrations alongside validated risk scores. We found that area under the receiver operating curve (ROC AUC) of the nominal regression model was highest for eotaxin when compared to sGPVI or conventional bleeding risk scores including ARC-HBR, PARIS bleeding, or PRECISE DAPT.

**Supplementary Figure S8.** Receiver operating characteristic (ROC) curves depicting the diagnostic accuracy of XGBoost model for patients with (BARC 2-5) and without (BARC <2) relevant bleeding events of the **(A)** test cohort and **(B)** entire study cohort. Areas under the curve of the validated machine learning algorithm unveils a good diagnostic accuracy of the model integrating important risk parameters including circulating chemokines.
